# A Framework for Assessing Import Costs of Medical Supplies and Results for a Tuberculosis Program in Karakalpakstan, Uzbekistan

**DOI:** 10.34133/2021/9813732

**Published:** 2021-08-25

**Authors:** Stefan Kohler, Norman Sitali, Nicolas Paul

**Affiliations:** ^1^Heidelberg Institute of Global Health, Faculty of Medicine and University Hospital, Heidelberg University, Heidelberg, Germany; ^2^Médecins Sans Frontières, Berlin, Germany; ^3^Institute of Social Medicine, Epidemiology and Health Economics, Charité–Universitätsmedizin Berlin, corporate member of Freie Universität Berlin and Humboldt-Universität zu Berlin, Berlin, Germany

## Abstract

*Background*. Import of medical supplies is common, but limited knowledge about import costs and their structure introduces uncertainty to budget planning, cost management, and cost-effectiveness analysis of health programs. We aimed to estimate the import costs of a tuberculosis (TB) program in Uzbekistan, including the import costs of specific imported items.*Methods*. We developed a framework that applies costing and cost accounting to import costs. First, transport costs, customs-related costs, cargo weight, unit weights, and quantities ordered were gathered for a major shipment of medical supplies from the Médecins Sans Frontières (MSF) Procurement Unit in Amsterdam, the Netherlands, to a TB program in Karakalpakstan, Uzbekistan, in 2016. Second, air freight, land freight, and customs clearance cost totals were estimated. Third, total import costs were allocated to different cargos (standard, cool, and frozen), items (e.g., TB drugs), and units (e.g., one tablet) based on imported weight and quantity. Data sources were order invoices, waybills, the local MSF logistics department, and an MSF standard product list.*Results*. The shipment contained 1.8 million units of 85 medical items of standard, cool, and frozen cargo. The average import cost for the TB program was 9.0% of the shipment value. Import cost varied substantially between cargos (8.9–28% of the cargo value) and items (interquartile range 4.5–35% of the item value). The largest portion of the total import cost was caused by transport (82–99% of the cargo import cost) and allocated based on imported weight. Ten (14%) of the 69 items imported as standard cargo were associated with 85% of the standard cargo import cost. Standard cargo items could be grouped based on contributing to import costs predominantly through unit weight (e.g., fluids), imported quantity (e.g., tablets), or the combination of unit weight and imported quantity (e.g., items in powder form).*Conclusion*. The cost of importing medical supplies to a TB program in Karakalpakstan, Uzbekistan, was sizable, variable, and driven by a subset of imported items. The framework used to measure and account import costs can be adapted to other health programs.

## 1. Introduction

Medical supplies (e.g., pharmaceutical products, diagnostic tests, or test reagents) are often procured on international markets and then imported. The World Trade Organization estimated that medical goods accounted for more than 5.3% of the global trade value in 2019, with more than half of that value coming from medicine trade [1]. Reasons to import medical supplies include limited in-country sales and distribution, limited production capabilities, rights or quality, or more efficient production elsewhere [2– 6]. The Global Fund, which supports health programs in low-resource settings, for instance, purchases health products only if these comply with quality standards (e.g., antituberculosis drugs prequalified under the World Health Organization Prequalification Programme) [7].

Health programs encounter import costs, which this study defined as international transport costs, customs clearance costs, and national transport costs, when they pay transport or customs-related charges for the procurement of medical supplies on international markets. Importing to low-resource or landlocked countries has been associated with higher costs than importing to other countries [8– 11], which potentially adds to the health financing challenges that low-resource countries face [12, 13]. Consistent with the notion that the import of medical goods can substantially increase their costs, World Health Organization guidelines recommend assessing international transport costs, import duties and subsidies, local transport costs, and distribution costs in cost-effectiveness analyses [14]. Other international organizations, like the United Nations Development Programme and Stop TB Partnership, request optimized packaging from supply partners to minimize freight costs in medical supply procurement [15, 16]. In addition to being of interest in program evaluation and medical supply procurement of donor agencies, import cost data can inform program planning and cost management. Knowing import costs and their composition may, for instance, improve budget calculations when program activities change, like a tuberculosis (TB) program expanding or shifting its focus from drug-susceptible to multidrug-resistant TB treatment.

Few studies to date have assessed import costs in health programs, to our knowledge, and none of these allocated import costs to specific imported medical supplies or suggested a framework for this process. Previous studies assessing import costs in a health program examined procurement data and/or conducted key informant interviews [17– 21]. Local transportation and distribution costs [22– 25], people’s cost to access a health program [26– 28], or sample collection costs in health programs [29– 32], all seem to have been more frequently assessed for health programs than import costs. Cost and cost-effectiveness analyses of health programs have ignored import costs or made ad hoc assumptions [33– 35], assessed billing records and/or asked key informants [19– 21], or assumed an average derived for a country [36– 38].

Country import costs have been estimated by comparing a country’s trade inflows at the cost, insurance, and freight (CIF) value with corresponding trade outflows of exporting countries at the free on board (FOB) value, assuming the difference between these values resembles international transport costs [39– 41]. Average markups for international transport of, for instance, 16%, 26%, and 49% have been estimated for imports to Denmark, the Russian Federation, and Burkina Faso, respectively [41]. CIF/FOB ratios are often the only available transport cost estimates, but they have been found to deviate considerably from directly measured international transport costs [42]. Furthermore, the CIF value excludes customs-related charges and transport costs within the destination country. Both limits the usefulness of CIF/FOB data for import cost assessment within program evaluation and planning. Direct measurement of import costs in a health program, as conducted in our study, overcomes these limitations and, in addition, allows to investigate the composition of import costs.

We applied costing and cost accounting principles to ordering and shipping information of a TB program in Karakalpakstan, Uzbekistan, to assess its average import costs and the import costs of individual items and units imported. Our broader aims are to share a framework for assessing import costs and to illustrate the type of information that import cost measurement and import cost accounting can generate.

## 2. Material and Methods

### 2.1. Study Setting and Design

Uzbekistan is landlocked and belongs to the 30 high multidrug-resistant TB burden countries [43]. Médecins Sans Frontières (MSF) has been supporting TB control in Karakalpakstan, a republic in Uzbekistan’s northwest, since 1998. The TB program in Karakalpakstan is an active research site and has been previously described [44– 46]. Medical supplies for the TB program are imported to Nukus, the capital of Karakalpakstan, from the MSF Amsterdam Procurement Unit in the Netherlands [47]. Typical shipments originate from a central storage in Amsterdam and are flown from Amsterdam to Tashkent. After customs clearance, cargo to the TB program in Karakalpakstan is transported by truck to a central storage in Nukus. Humanitarian goods are exempted from import duties in Uzbekistan, but the TB program encounters customs clearance costs when cargo is kept in interim storage rented by MSF in Tashkent until it is cleared by the customs authorities, which can take several weeks, and when the TB program hires a customs agent to declare the imported item lines. 

**Figure 1:**
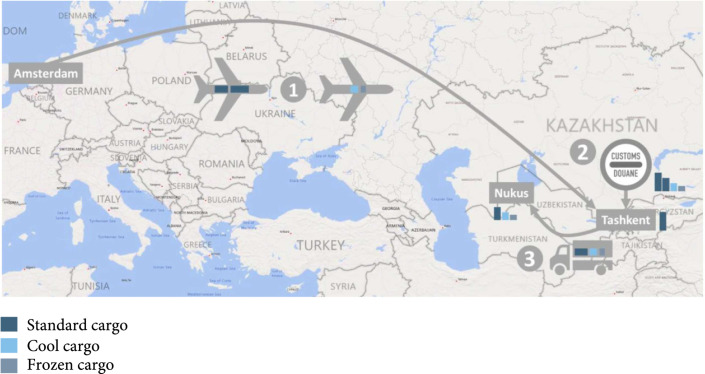
Transport route, customs point, and means of transport for medical supplies imported by a tuberculosis program in Uzbekistan. Import costs to a tuberculosis program in Nukus were assessed for a shipment of medical supplies that contained standard cargo, cool cargo, and frozen cargo. Cargos drawn in the same plane or truck were transported and billed together. Only cargo shipped to Nukus caused import costs for the TB program in Karakalpakstan.

We studied the transport and customs clearance costs of one major shipment of drugs and laboratory items to the TB program in Karakalpakstan. The shipment, which arrived by truck in Nukus on December 29, 2016, combined 18 individual orders and consisted of three different types of cargo (standard, cool, and frozen). The three different cargos were flown from Amsterdam to Tashkent via Frankfurt on two separate flights in November and December 2016. The standard cargo was composed of two parts: one part was forwarded to Nukus, the other part remained in Tashkent. The cargo part remaining in Tashkent was excluded from import cost assessment (Figure 1 and supplementary Table S1). 

### 2.2. Data Sources

Order invoices and waybills for air and road transport were obtained from the local MSF logistics department. Order invoices contained information about ordered items, including a short item description, ordered quantities, item lines, and unit prices at the time of ordering. Separate air waybills were available for each cargo type and contained the total air freight cost, cargo gross weight, cargo volume, the chargeable weight (i.e., the maximum of gross weight and volumetric weight), the rate charged by kilogram chargeable weight, and surcharges. An MSF waybill for the in-country road transport included a packing list as well as the gross weight and volume of each cargo. Road transport cost for the shipment and an estimate of the customs clearance costs per item line were gathered through written and oral communication with MSF logistic coordinators in Tashkent, who coorganized MSF shipments to Uzbekistan.

Average weight for one unit of an item (e.g., 0.9 g for one tablet of the antibiotic pyrazinamide packaged in a blister of 672 units) was extracted from the MSF Green List for all ordered items. The MSF Green List is a standard product list of MSF for frequently used and ordered medical items. It is similar to the Global Drug Facility Product Catalog and contains information about an item’s active ingredient, pharmaceutical form, and packaging type and size, as well as unit weights, volumes, and prices [48, 49]. 

Orders and air waybills were issued in Euro (€); customs clearance charges were estimated in US dollar ($); land freight costs in Uzbekistani Som (UZS). All currencies were converted to Euro using World Bank annual exchange rates for the year 2016: €1=$1.107=UZS 3282 [50]. 

### 2.3. Import Cost Assessment Framework

To assess import costs in the TB program, we derived the following steps from general principles of costing and cost accounting (also called cost assignment; see, e.g., [51, 52]): 

*Step 1*. Description of the import process and identification of the causes of import costs from the perspective (or viewpoint) assumed in the cost assessment 

*Step 2*. Collection and inspection of data on import costs for at least one shipment and cargo type 

*Step 3*. Choice of a cost allocation base for each import cost 

*Step 4*. Allocation of import cost totals in proportion to the allocation bases:

(a) To parts of a shipment with different final destinations
(b) To different cargo types in a shipment
(c) To units in a shipment

*Step 5*. Calculation of allocated import cost totals (and repetition of steps 4–5 until reaching step 4c) 

Steps 1–2 guide import cost measurement, whereas steps 3–5 guide the allocation of import cost totals to parts of a shipment. We describe next how we applied these steps to a shipment of medical supplies to a TB program:

*Steps 1*. The import process to the TB program was discussed with staff who were familiar with the procurement process. Afterwards, a simplified import process that includes the presumed main causes of import costs (air freight, customs-related, and land freight) was described (Figure 1). 

*Steps 2–3*. Import cost data were collected (see 2.2 Data Sources) and inspected for variable and fixed costs to understand how cargo characteristics determine import costs. Air freight charges included per-kg costs and fixed costs. We chose weight as the cost allocation base. For land freight, a truck was rented at a fixed cost and, hence, no characteristic of the imported items correlated with land freight costs. Again, we chose weight as the cost allocation base. Customs-related costs excluded customs duties, as humanitarian goods were imported, but involved customs clearance costs. For customs clearance, customs agent fees were paid per customs declaration and number of pages of each declaration. We chose the units per cargo and item line as the cost allocation base for the estimated semifixed customs clearance cost. 

**Figure 2:**
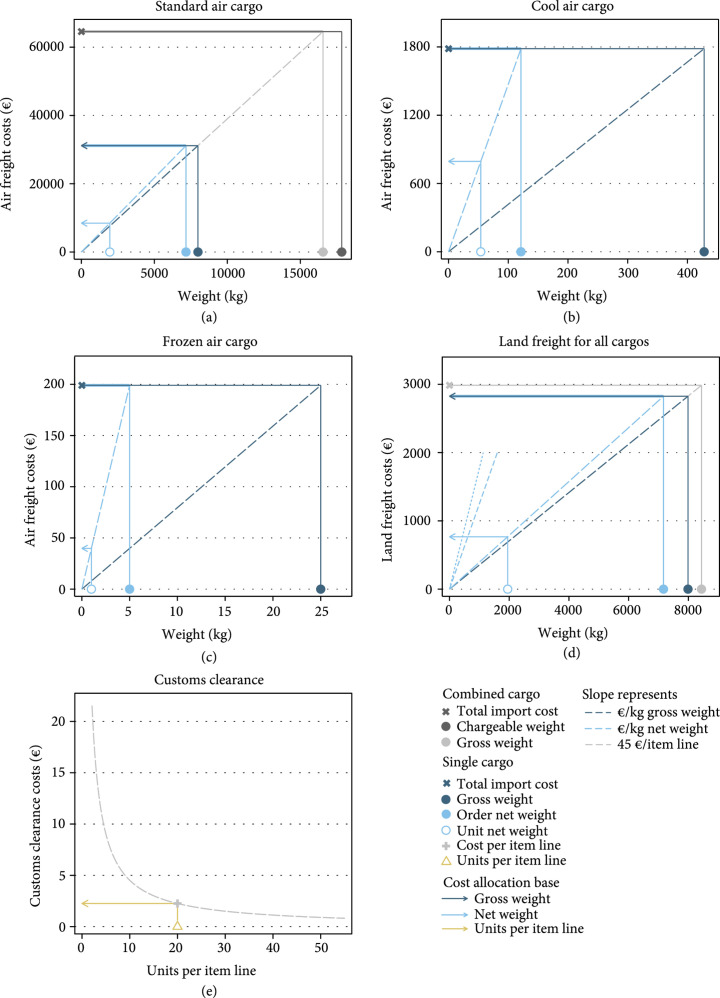
Graphical representation of import cost accounting. Shown values for unit net weights and units per item line exemplify how unit import costs were generated through cost allocation. Import costs allocated to standard cargo units and items are shown in Figures S1- 2. (a) Standard air cargo. Standard air cargo contained two parts. One part was forwarded to the TB program. (b) Cool air cargo. (c) Frozen air cargo. (d) Land freight for all cargo. Land freight to the TB program contained standard, cool, and frozen cargo. Cost allocation is illustrated for standard cargo. €/kg net weight is shown for standard cargo (long dashed line), cool cargo (medium-long dashed line), and frozen cargo (short dashed line). (e) Customs clearance.

*Step 4a*. The standard air cargo contained two parts, of which one part remained in Tashkent and the other part was forwarded to Nukus. As only the cargo shipped to Nukus caused import costs from the perspective of the TB program in Karakalpakstan, the air freight cost for the standard cargo part shipped to Nukus was estimated by splitting the total amount billed proportionally to the gross weight distribution between the two cargo parts (grey and dark blue lines in Figure 2(a)). 

*Step 4b*. Standard cargo for the TB program in Karakalpakstan was transported to Nukus in a truck together with the cool and frozen cargo. The land freight cost was allocated to the three cargos proportionally to their gross weight (grey and dark blue lines in Figure 2(d)). 

*Steps 4c*. For each cargo type, the transport cost totals were allocated to units by multiplying unit weight (i.e., the weight of one unit of an item) with the average cost of transporting the net weight. Average net weight transport costs were calculated by dividing total transportation costs by the order net weight for each cargo (light and dark blue lines in Figures 2(a)– 2(d)). Order net weight excluded the weight of cargo-specific packaging. It was estimated by multiplying unit weights with ordered quantities and summing-up over all items in a cargo. Unit customs clearance costs, in turn, were calculated separately for each cargo by dividing the estimated customs clearance costs of €45 per item line by the number of units imported within a line (Figure 2(e)). 

*Step 5*. Import cost totals were calculated for each cause of import costs, each cargo type, and the whole shipment. Air freight costs of the cool and frozen cargos shipped to the TB program corresponded to the total charges invoiced. Other transportation cost for cargo had to be obtained through cost allocation steps 4a and 4b. Total customs clearance costs were calculated by multiplying the number of item lines in a cargo with the estimated customs clearance costs of €45 per item line. The total import cost of the shipment to the TB program was calculated by adding up the import costs of all cargos delivered to Nukus. 

### 2.4. Data Analysis

Import cost accounting generated unit import costs for each of the 85 imported items (e.g., the cost of importing 1 tablet levofloxacin). Item import costs (e.g., the cost of importing an order quantity of 1200 tablets levofloxacin) were calculated by multiplying the unit import cost and the quantity for the imported items. Measured and generated import costs were described using univariate statistics as well as bar, surface, and box plots and histograms. In bivariate analyses, items were grouped by cargo type (standard, cool, and frozen), by cargo type and pharmaceutical form (capsule, fluid<100 ml, fluid≥100 ml, tablet, ointment, powder, test/test kit, and wipe), and by cargo type and item packaging (ampulla, bottle, capsule or tablet, fluid bag, sachet, tube, vial, and test/test kit). Fluids belonging to a test or test kit were included in the test/test kit group. To compare the equality of the distributions of continuous variables across item groups, Kruskal-Wallis tests were applied. For multiple pairwise comparisons following a Kruskal-Wallis test, Dunn’s test with Bonferroni adjustment was applied. Shapiro-Wilk tests were applied to evaluate normal distribution of data. Regression models with log 10-transformed continuous variables (i.e., unit import cost, weight, and price) were used to assess how much of the variation in the unit import costs is explained by the following item characteristics or combinations thereof: unit weight, unit price, pharmaceutical form, item packaging, and cargo type. 

Import costs were analyzed in absolute terms and relative terms (% of the imported value). For two items in the shipment with unit prices that did not reflect the item values, no percentage import costs were calculated: The first item, a reagent set, was shipped as frozen cargo and was freely available to the TB program through the Stop TB Partnership. The second item was a test kit consisting of two parts that were separately shipped as cool and frozen cargos. While the full item price of the test kit was reported on the invoice for cool cargo, only a nominal unit price of €0.01 was reported on the frozen cargo invoice. Multiple orders of the same item were combined before analysis by summing up ordered quantities and determining a quantity-weighted average price across orders. Unless specified otherwise, net weight (including pharmaceutical packaging) rather than gross weight (including pharmaceutical packaging and cargo packaging) is reported. Statistical significance was assumed at P<0.05. All analyses were performed in Stata 15.1 SE. 

### 2.5. Cost Assessment Assumptions

For all import costs that were allocated, an assumption about what constitutes a reasonable allocation base was made. A reasonable cost allocation base would be an item characteristic for which data is available and which correlates strongly with the import cost of the TB program. Latter property can be difficult to assess and to fulfill. For instance, while the largest portion of the air freight costs (64–84%) was charged based on chargeable weight, of which 93% to 100% was gross weight, only 20% to 90% of the gross weight was net weight. The land freight cost was charged lump-sum without any indicative cost breakdown. Yet, we chose gross weight and net weight as allocation bases for all transport costs.

Unit weight was not available for 6 of 69 standard cargo items, 2 of 14 cool cargo items, and 2 of 2 frozen cargo items. For standard and cool cargo items, four missing unit weights of drugs were substituted with available unit weights of the same active ingredient in a similar concentration after reviewing the plausibility of this substitution based on item concentration and packaging; the other four missing values were replaced by the median unit weight of similarly packaged items. The same unit weight was assumed for the frozen cargo items, which is equivalent to allocating import costs based on the imported number of units. Item lines were defined using Harmonized Commodity Description and Coding System (HS) codes reported in the order invoices.

Transportation from manufacturers to a central storage of the MSF Procurement Unit in Amsterdam, storage in Amsterdam, transportation from the central storage to the airport in Amsterdam, storage in Nukus, and transportation from the central storage in Nukus to clinics in Karakalpakstan (i.e., the point of care) are part of the supply chain of the TB program. Costs for these parts of the supply chain, indirect import costs (e.g., administrative costs or disposal of cargo packaging), or nonfinancial import costs (e.g., the lead time to import) were excluded from this import cost assessment.

## 3. Results

### 3.1. Shipment Characteristics

The studied shipment contained standard, cool, and frozen cargo for a TB program in Karakalpakstan. It combined 18 orders for medical supplies from the MSF Procurement Unit in Amsterdam. In total, over 1.8 million units (e.g., a single tablet or test) of 85 items of pharmaceutical and diagnostic supplies (e.g., antibiotics or test reagents) were delivered jointly to a central storage of the TB program in Nukus. Unit value (€0.003–289) and weight (0.1 g–1.5 kg) varied within and across cargos. Over 95% of the imported gross weight and 98% of the imported net weight resulted from the standard cargo. The number of item lines to be declared by the customs agent ranged from one line of items in the frozen cargo to seven lines in the standard cargo. Most standard cargo items were delivered in higher quantity and had less weight and lower unit prices than the cool or frozen cargo, which contained tests or test kit materials and insulin vials. The ordered items were estimated to weigh, net of cargo packaging, 7168 kg in the standard cargo, 121 kg in the cool cargo, and 5 kg in the frozen cargo. Corresponding cargo gross weights that include cargo packaging exceeded the estimated net weights by factors of 1.1, 3.5, and 5, respectively (Table 1). 

**Table 1:** Characteristics of a shipment of medical supplies to a tuberculosis program in Uzbekistan.

	Standard cargo	Cool cargo	Frozen cargo
Order			
Orders combined	10	5	3
Items	69	14	2
Item lines	7	3	1
Units	1,840,928	1218	100
Units per item^∗^	4000 (450–24,000)	4 (2–55)	50 (20–80)
Units per item line	35,000 (8000–595,172)	55 (20–1143)	100
Unit price (€)^∗^	0.10 (0.03–0.58)	26 (9.35–57)	24 (0.01–45)
Unit weight (g) ^∗^	1.2 (0.7–15)	126 (100–550)	50
Order net weight (kg)	7168	121	5
Order value (€)	385,167	20,228	901

Cargo			
Gross weight (kg)	7987	428	25 (20 dry ice)
Gross-to-net weight ratio	1.11	3.53	5

Median (IQR).^∗^Kruskal-Wallis test rejects the equality across cargo types (P<0.001). Handling information: standard $\text{cargo}=\text{store }15–25^{{}^{\circ}}\text{C}$, cool cargo=keep cool 2–8°C and do not freeze, and frozen cargo=1 piece with dry ice (UN1845). Order net weight and value were estimated by multiplying the unit weight and unit price, respectively, with the ordered quantity and summing-up over the items in a cargo. The same unit weight was assumed for frozen cargo items.

### 3.2. Total and Average Import Costs

**Figure 3:**
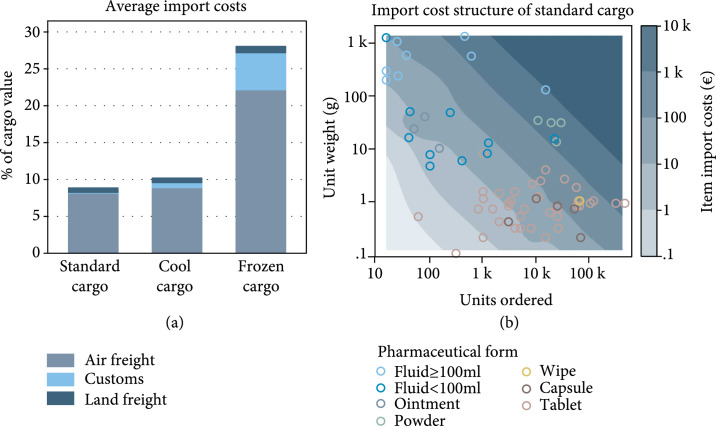
Import costs of medical supplies for a tuberculosis program in Uzbekistan. (a) Average import costs. (b) Import cost structure of standard cargo. Each circle represents one of the 69 standard cargo items. Background colors represent how much of the total import cost, including transport and customs clearance costs, has been attributed to the item. Import costs between circles are interpolations.

Air freight costs varied by cargo type (€199–31,155 per cargo) due to different cargo gross weights, freight rates, and surcharges. Estimated customs clearance costs accumulated to €45 to €316 for the different cargos. Land transport costed €2985 for the truckload, of which €9 to €2826 were allocated to each cargo based on gross weight. The total import cost amounted to €34,297 (8.9% of the order value) for the standard cargo, €2072 (10%) for the cool cargo, €253 (28%) for the frozen cargo, and €36,621 (9.0%) for the whole shipment. The average import cost per kg net weight was €4.78 for the standard cargo, €17 for the cool cargo, €51 for the frozen cargo, and €5.02 for the whole shipment. The differences in the average import cost per kg net weight across cargos reflect different air freight rates and fuel surcharges, different amounts of weight added to the order net weight by cargo packaging (e.g., 20 out of 25kg dry ice), and a varying number of units per imported item line and weight (Table 2 and Figure 3(a)). 

**Table 2:** Costs of importing medical supplies to a tuberculosis program in Uzbekistan.

	Standard cargo	Cool cargo	Frozen cargo
Import costs (€)			
Air freight	31,155	1785	199
Customs clearance	316	136	45
Land freight	2826	151	8.84
Total	34,297	2072	253

Average import costs			
Air freight (€/kg net weight)	4.35	15	40
Customs clearance (€/item line)	45	45	45
Land freight (€/kg net weight)	0.39	1.25	1.77
Total (€/kg net weight)	4.78	17	51

Unit import costs (€/unit)^∗^			
Air freight	0.005 (0.003–0.06)	1.85 (1.47–8.09)	1.99 (1.99–1.99)
Customs clearance	0.00008 (0.00008–0.00008)	0.04 (0.04–0.04)	0.45 (0.45–0.45)
Land freight	0.0005 (0.0003–0.006)	0.16 (0.16–0.69)	0.09 (0.09–0.09)
Total	0.007 (0.003–0.07)	2.16 (1.80–8.98)	2.53 (2.53–2.53)

Percentage unit import cost (% of unit price) ^†^			
Air freight	11 (4.2–27)	5.9 (3.1–16)	4.4 (4.4–4.4)
Customs clearance	0.08 (0.01–0.3)	0.2 (0.07–0.4)	1.0 (1.0–1.0)
Land freight	1.0 (0.4–2.4)	0.5 (0.3–1.3)	1.3 (0.2–2.4)
Total	12 (4.6–35)	8.7 (3.8–21)	5.6 (5.6)

*N* = ^∗^ 85 and ^†^83. Median (IQR). Percentage import costs exclude a donated item in the cool cargo and the frozen part of a test kit with unit prices of €0 and €0.01. Kruskal-Wallis tests reject equality of the unit import cost distribution across cargo types for air freight, customs clearance, land freight, and total import costs (all P<0.001). Equal distribution across cargo types was not rejected for percentage unit import costs (air freight P=0.46, customs clearance P=0.12, land freight P=0.24, and total unit import cost P=0.67). Unit import cost distributions are shown in supplementary Figures S3–4 and summarized in Tables S2–4.

### 3.3. Unit Import Costs

Total import cost allocation generated unit import costs. The median unit import cost was €0.007 (interquartile range (IQR) 0.003–0.07) for standard cargo items, €2.16 (IQR 1.80–8.98) for cool cargo items, and €2.53 (IQR 2.53–2.53) for frozen cargo items (Kruskal-Wallis test P<0.001). Related to, on average, higher unit prices of cool and frozen cargo items, median unit import costs as a percentage of unit prices were more similar than absolute unit import costs across standard cargo items (median 12%, IQR 4.6–35), cool cargo items (8.7%, IQR 3.8–21), and frozen cargo items (5.6%, IQR 5.6–5.6, P=0.67). 

**Figure 4:**
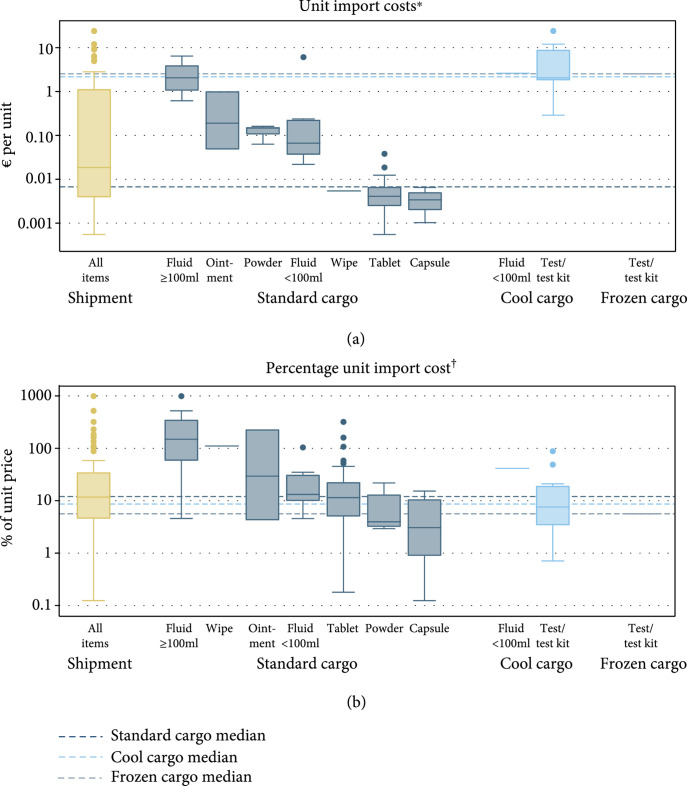
Unit import costs of medical supplies for a tuberculosis program in Uzbekistan, by pharmaceutical form of imported items. *N* = ^∗^85 and ^†^83. Logarithmic y-axis. Tablets (38 items), fluids (19 items), test or test kit materials (15 items), capsules (5 items), powder (4 items), ointments (3 items), and wipe (1 item). Unit import costs by item packaging are shown in supplementary Figure S5. (a) Unit import costs^∗^. (b) Percentage unit import cost ^†^. Percentage unit import costs exclude a donated item and the frozen cargo part of a test kit that was split across cargos.

Figure 4 shows the variation in absolute and percentage unit import costs for all medical supply items in the shipment and by items’ cargo type and pharmaceutical form. Median unit import cost ranged from €0.003 per capsule imported as standard cargo to €2.61 per 10 ml vial of insulin imported as cool cargo. For comparison, median unit import cost as a percentage of the unit price ranged from 3.1% per capsule imported as standard cargo to 149% per fluid≥100 ml imported as standard cargo. Comparing standard cargo items only, the median import cost of €2.06 per unit of fluid≥100 ml was over 600 times more than the median import cost of €0.003 for a capsule. In terms of percentage unit import costs, a more than 40-fold import cost difference remained (149% versus 3.1%). 

Regression analyses indicate that knowing the cargo type of an item as well as its pharmaceutical form or packaging would have given a good indication of the magnitude of an item’s unit import cost. Approximately 90% of the variation in the log-transformed unit import cost is explained by the cargo type and either an imported item’s pharmaceutical form or packaging. Cargo type and knowing if an item contains fluids ≥ 100 ml explained 69% of the variation in unit import cost, similar to knowing an item’s cargo type and unit price (67%). Percentage unit import costs (IQR 4.5–35%) varied less than unit import costs (IQR €0.004–1.14) and were predicted with lower explanatory power. Knowing an item’s price, its cargo type, and its pharmaceutical form or packaging would have explained 72% and 75% of the variation in the percentage unit costs of the imported medical supplies, respectively (supplementary Tables S5–6). 

### 3.4. Import Cost Structure

Item import costs were calculated by multiplying order quantities and unit import costs. Figure 3(b) illustrates the contribution of individual order items to the total import cost of the standard cargo. Circles represent items. The position of a circle shows the item import cost. The color of a circle indicates the pharmaceutical form of an item. Items closer to the upper right corner were associated with higher import costs than items closer to the lower left corner. The highest cost band (€1000–10,000) contains fluids, powders, and two types of antibiotic tablets. Cost accounting associated €29,057 (85%) of €34,297 of the standard cargo import cost with the items found in the highest import cost band. These items represented ten (14%) of 69 standard cargo items and 901,879 (49%) of 1.8 million ordered units. The item associated with the highest import costs in the standard cargo was 15,000 units of 0.9% sodium chloride solution in a 100 ml flexible bag, to which €9246 (27%) of the standard cargo import cost was attributed. 

### 3.5. Import Cost Contributors

Due to using net weight as the transport cost allocation base and the fact that 99% of the standard cargo import cost to the TB program was related to transport, most imported items contributed to the costs of importing through their net weight. Figure 3(b) suggests that four groups of imported items can be distinguished within the standard cargo as follows:

(1) “Costly by number” items with relatively low unit weights but ordered in higher numbers (approximately lower-right quadrant)
(2) “Costly by unit weight” items ordered in lower numbers but with relatively high unit weights (approximately upper-left quadrant)
(3) “Costly by unit weight × number” items with medium-high unit weights and ordered in medium-high numbers (approximately upper-right quadrant)
(4) “Low import cost” items with relatively low unit weights and ordered in relatively low numbers within an item line already existing in the cargo (approximately lower-left quadrant)

All but one “costly by number” items were tablets or capsules. They contributed to the imported weight, and thus import costs, predominantly through the ordered quantity (e.g., pyrazinamide or isoniazid/rifampicin fixed-dose combination tablets). Most imported fluids, particularly large-volume fluids, in turn, were “costly by unit weight” items, which cause higher import costs than other items already when ordered in low quantity due to the higher weight of each unit (e.g., 500 ml Ringer’s lactate or 1 l sodium chloride solutions). “Costly by unit weight × number” items were items in powder form and some fluids. While on average lighter than the “costly by unit weight” items and ordered in lower quantity than “costly by number” items, the “costly by unit weight × number” items contributed to import costs through their combination of unit weight and the quantity ordered (e.g., 15,000 units of 0.9% sodium chloride solution in a 100 ml flexible bag). The six “costly by unit weight × number” items in the shipment were associated with €21,179 (62%) of €34,297 of the standard cargo import costs. Finally, some items had low unit weight, were ordered in low quantity, and had low per-unit customs clearance costs due being imported within an item line that, overall, contained a high number of units (e.g., risperidone and levothyroxine sodium tablets). We classified these items as “low import cost” items.

## 4. Discussion

### 4.1. Summary of Findings

Import costs are context-specific and cargo-specific, and they can be substantial relative to the value of an imported good [8– 10]. Applying general costing and cost accounting principles, we assessed the transport and customs-related costs of a major shipment from the MSF Procurement Unit in Amsterdam, the Netherlands, to a TB program in Karakalpakstan, Uzbekistan. 

We estimated that the total import cost of the shipment was 9.0% of its value. The largest portion of the TB program’s total import cost was related to transport (82–99% of a cargo’s import cost). Import costs varied when assigned to the different types of cargo (8.9%, 10%, and 28% of the standard, cool, and frozen cargo value, respectively) or to the different units and items imported (median 12% of the imported value, IQR 4.5–35). While import costs were expected to differ by cargo type, we were surprised by how much more the allocated import costs varied between items and units within the same cargo type.

The ten items that were most costly to import in the standard cargo were associated with 85% of the standard cargo import costs. Import cost contributors could be categorized in four groups: (1) “costly by number” items, (2) “costly by unit weight” items, (3) “costly by unit weight × number” items, and (4) “low import cost” items. The relatively large contribution of “costly by unit weight × number” items to import costs might have been the most difficult to detect without import cost accounting, as it resulted from a combination of two item characteristics. In turn, additional “low import cost” items could have been added for a small additional cost to the shipment, as their per-item transport costs and customs clearance costs would have been relatively low.

### 4.2. Comparison with Previous Findings

Studies that assessed import costs on a country level based on trade flows reported higher international transport costs to landlocked and low-resource countries than for other countries [9, 53]. Hence, especially health programs in low-resource countries can be at risk for a high dependency on import of medical supplies in a context of high import costs. Despite their potential relevance for program planning and evaluation, we found few studies that assessed import costs or similar costs (e.g., international transport costs, procurement costs, or supply chain costs) in health programs [17– 20]. One study interviewed procurement officers in two international organizations and a nongovernmental organization about the import costs to countries with deworming programs. The costs for international transport and customs of the deworming drug were estimated at 10% of drug value [20]. More similar data to ours has been assembled by the Global Fund, which regularly publishes reference air and ocean freight costs based on its transactional data. In the first quarter of 2021, for instance, median air freight costs of 16% (IQR 8–39) of the item value were reported for antiretroviral drugs, 30% (IQR 16–53) for antimalarial medicines, 17% (IQR 10–30) for HIVrapid diagnostic tests, and 42% (26–91) for malaria rapid diagnostic tests [18]. In contrast to our analysis of various items and diverse cargo that were imported by one TB program, the Global Fund analyzes the procurement of similar items for different programs. 

### 4.3. Practical Implications

Knowledge and understanding of the import costs of medical supplies can assist in health program evaluation, planning, and management. Applications for import cost assessment include:

(1) Measuring import costs to quantify the total import cost of a health program
(2) Assessing the full costs of purchasing and importing specific medical supplies based on unit import costs. Using the presented framework and results, we estimated in another study that one course of TB treatment can require the import of 0.6 kg to 36 kg of drugs, drug packaging, and cargo packaging. Importing this weight to the TB program in Karakalpakstan could add between $3.16 and $185 to the purchasing cost of one drug regimen with import costs updated to 2020/21 prices and converted to US dollar [54]
(3) Unveiling medical supplies to which relatively high import costs are allocated. While cost allocation relies on assumptions, its results can be a sound starting point for further investigation
(4) Optimizing the packaging of imports to reduce waste and import costs (compare [16, 55]). Import cost assessment quantifies the difference between cargo net and gross weight, which is indicative for the transport costs of packaging
(5) Identifying high-cost contributors among imported supplies. If alternative international transport options were available, suitable (e.g., timely, safe, reliable), and feasible at a reasonable administrative costs for a health program, then two-tier international transport (e.g., air shipping for time-critical, volatile supplies, and land/maritime shipping otherwise) could be used to save import costs. Developing local supply sources or production of items that cause high import costs might be another opportunity for supply chain optimization, especially for long-term programs (see also [6])

For health programs that operate in low-resource settings, assessing and optimizing import costs may help to reduce a potential triple burden of costly import, a high level of dependency on imported medical supplies, and a large health financing gap. As import cost assessment itself is costly, mainly through personnel time spent on such assessments, systematic import cost measurement could be performed as a first step. If import cost measurement indicates import costs that are considered sizable and import processes that are modifiable, then import cost allocation could be performed as a second step.

### 4.4. Adaptivity of the Import Cost Assessment Framework

The presented framework to assess the import costs of the TB program in Uzbekistan describes general principles that can be applied to other health programs. Going through the five framework steps will indicate which import costs to measure and how to allocate them to specific units, also when the import process differs from our example (e.g., land transportation only, transportation via rail or sea freight, multiple final destinations, cargo subject to customs duties, or different customs clearance costs). While we used unit weights from the MSF Green List, such unit weights can be self-generated through weighing an item (once in stock) and dividing the weight by the number of units an item contains. Manufacturers or distributors may also be able to provide unit weight data. On another practical note, we received all required data from the local MSF logistics department. For import cost assessment in other health programs, data collection might require consulting several administrative units, suppliers, or manufactures; or data collection might rely more on key informant interviews (e.g., if import bills and order invoices are unavailable or if informal payments or nonfinancial costs, like import delays, are identified as main import cost causes).

### 4.5. Limitations of the Framework and Case Study

As allocated import costs are generated data, which are subject to assumptions and limitations, we see their value in pointing to differences in magnitude (e.g., import costs of 1% vs. 10% vs. 100% of the value of an imported good) rather than in their exact quantitative interpretation.

Specific limitations include that we assessed the financial import costs of drugs and laboratory items in only one major shipment to a TB program. The shipment allowed to assess three typical cargo types, but we could not evaluate if the analyzed mix of imported items or the utilization of the rented truck, which codetermined the allocated import costs, were similar to other shipments to the program. In addition, the precision of the unit weights was variable and uncertain, and some unit weights were missing and imputed. Imprecise unit weights could have introduced unknown error to the derived unit and item import costs.

Data was collected in late 2016. Import costs relative to import value could be robust estimates if costs and prices change similarly. However, shortly after data collection, the Uzbekistani Som substantially devalued against the US dollar and the Euro [50]. Due to the exchange rate change, the relative cost of land freight for the TB program may have decreased since the time of data collection. On the other hand, the COVID-19 pandemic may have raised the TB program’s international transport cost, at least temporarily, as a reduction in cargo capacity in passenger flights during the pandemic resulted in a global increase in air freight costs [56]. Continuous import cost assessment would allow to address several of these limitations. 

Notwithstanding challenges that can be addressed through collecting more or better data, the cost accounting also included normative choices. To assign import costs to individual items, we used the item net weight for air and land transport costs and the number of units per item line for customs clearance costs as allocation bases. Further, certain elements of import costs were not included in this study, such as the transport of goods to the exporting airport, optional freight insurance, storage at the final destination, distribution from the local warehouse to the point of care, administration, import time delays, or lead time. Customs clearance at Tashkent airport required, for instance, 36 days for the standard cargo and 21 days for the cool cargo.

## 5. Conclusion

Import cost measurement and accounting for a TB program in Karakalpakstan, Uzbekistan, suggested average import cost of 9.0% of the imported value and a broad variation in the import costs of specific medical supply items (median 12% of the imported value, IQR 4.5–35). The uneven distribution of import costs, which import cost accounting generated, exemplified how cost accounting provides detailed import cost information that can be used in program evaluation, planning, and cost management.

We found import costs to be an important yet understudied cost of health programs. Therefore, we hope that the presented framework and results from Uzbekistan will encourage others to assess import costs and share their findings. Health programs are in principle well-positioned to assess their own transportation and customs-related costs based on procurement data.

## Data Availability

The data and codes that support the findings of this study are openly available in heiDATA at doi: 10.11588/data/JM2H6I [57].

## Supplementary Materials

Supplementary Materials Table S1: Order and shipping details of medical supplies imported by a tuberculosis program in Uzbekistan in 2016. Table S2: Unit air freight, customs clearance, and land freight costs of medical supplies for a tuberculosis program in Uzbekistan. Table S3: Unit import costs of medical supplies for a tuberculosis program in Uzbekistan, by pharmaceutical form of imported items. Table S4: Unit import costs of medical supplies for a tuberculosis program in Uzbekistan, by packaging of imported items. Table S5: Predictors of unit import cost of medical supplies for a tuberculosis program in Uzbekistan. Table S6: Predictors of percentage unit import cost of medical supplies for a tuberculosis program in Uzbekistan. Figure S1: Unit import costs of medicines imported as standard cargo by a tuberculosis program in Uzbekistan, by item. Figure S2: Percentage unit import costs of medicines imported as standard cargo by a tuberculosis program in Uzbekistan, by item. Figure S3: Distribution of the unit import costs for a tuberculosis program in Uzbekistan. Figure S4: Distribution of the percentage unit import costs for a tuberculosis program in Uzbekistan. Figure S5: Unit import costs of medical supplies for a tuberculosis program in Uzbekistan, by packaging of imported items.
