# Supplementary material for: A Framework for Assessing Import Costs of Medical Supplies and Results for a Tuberculosis Program in Karakalpakstan, Uzbekistan
Source: Health Data Sci. 2021 Aug 25;2021:9813732. doi: 10.34133/2021/9813732 (PMC10904066; doi:10.34133/2021/9813732)
Supplement: Supplementary Materials — Table S1: Order and shipping details of medical supplies imported by a tuberculosis program in Uzbekistan in 2016. Table S2: Unit air freight, customs clearance, and land freight costs of medical supplies for a tuberculosis program in Uzbekistan. Table S3: Unit import costs of medical supplies for a tuberculosis program in Uzbekistan, by pharmaceutical form of imported items. Table S4: Unit import costs of medical supplies for a tuberculosis program in Uzbekistan, by packaging of imported items. Table S5: Predictors of unit import cost of medical supplies for a tuberculosis program in Uzbekistan. Table S6: Predictors of percentage unit import cost of medical supplies for a tuberculosis program in Uzbekistan. Figure S1: Unit import costs of medicines imported as standard cargo by a tuberculosis program in Uzbekistan, by item. Figure S2: Percentage unit import costs of medicines imported as standard cargo by a tuberculosis program in Uzbekistan, by item. Figure S3: Distribution of the unit import costs for a tuberculosis program in Uzbekistan. Figure S4: Distribution of the percentage unit import costs for a tuberculosis program in Uzbekistan. Figure S5: Unit import costs of medical supplies for a tuberculosis program in Uzbekistan, by packaging of imported items. [file 9813732.f1.docx]

**Supplementary material**

**Table S1:** Order and shipping details of medical supplies imported by a tuberculosis program in Uzbekistan in 2016

|  | **Standard cargo**  **(part 1)** | **Standard cargo**  **(part 2)** | **Cool cargo** | **Frozen cargo** |
| --- | --- | --- | --- | --- |
| Origin | MSF Procurement Unit Amsterdam | | | |
| Destination | MSF Tashkent | MSF Nukus (TB program Karakalpakstan) | | |
|  | | | | |
| *Order* | | | | |
| Orders combined |  | 10 | 5 | 3 |
| Items |  | 69 | 14 | 2 |
| Item lines |  | 7 | 3 | 1 |
| Units |  | 1,840,928 | 1218 | 100 |
| Units per item |  | 4000  (450–24,000)  [15–465,696] | 4  (2–55)  [2–960] | 50  (20–80)  [20–80] |
| Units per item line |  | 35,000  (8000–595,172)  [50–1,107,506] | 55  (20–1143)  [20–1143] | 100  (100–100)  [100–100] |
| Unit price (€) |  | 0.10  (0.03–0.58)  [0.003–62] | 26  (9.35–57)  [0–289] | 23  (0.01–45)  [0.01–45] |
| Unit weight (g) |  | 1.2  (0.7–15)  [0.1–1356] | 126  (100–550)  [15.6–1500] | 50  (50–50)  [50–50] |
| Order weight |  | 7168 | 121 | 5 |
| Order value |  | 385,167 | 20,228 | 901 |
|  | | | | |
| *Air freight cargo (Amsterdam to Tashkent)* | | | | |
| Pieces | 1486 | | 9 | 1 |
| Volume (m^3^) | 107.018 | | 2.544 | 0.064 |
| Gross weight (kg) | 16,553.5 [8566.2+7987.3] | | 428 | 25 (20 dry ice) |
| Chargeable weight (kg) | 17,836.5 [9230.1+8606.4] | | 428 | 25 |
| Freight rate (€/kg) | 2.97 | | 3.52 | 5.11 |
| Other charges (€) | 11,594 | | 278 | 71.25 |
| Total costs (€) | 64,568 [33,413+31,155] | | 1785 | 199 |
|  | | | | |
| *Customs (Tashkent)* | | | | |
| Customs clearance cost (€/line) | 45 | | | |
|  | | | | |
| *Land freight cargo (Tashkent to Nukus)* | | | | |
| Pieces |  | 491 | 9 | 1 |
| Volume |  | 28,924 | 2544 | 7987 |
| Gross weight (kg) |  | 7987.3 | 428 | 25 |
| Total costs (€) |  | 2985 | | |

Median (IQR) [range]. [Tashkent+Nukus parts]. MSF = Médecins Sans Frontières. Handling information: standard cargo = store 15–25°C, cool cargo = keep cool 2–8°C and do not freeze, frozen cargo = 1 piece with dry ice (UN1845). Chargeable weight is the maximum of gross weight and volumetric weight. Other charges were fuel surcharges plus, for frozen cargo, dangerous good fee. Cool cargo contained an item made available through the Stop TB Partnership at no charge. Frozen cargo contained the frozen part of a two-part test kit to which a unit price of €0.01 was assigned. Unit weights are based on the MSF Green List. Missing unit weights for 6 of 69 items in standard cargo and 2 of 14 items in cool cargo were imputed using the median unit weight of comparable items. The same unit weight was assumed for all frozen cargo items.

**Table S2:** Unit air freight, customs clearance, and land freight costs of medical supplies for a tuberculosis program in Uzbekistan

| **Import cost** | **Cargo** | **Median** | **Interquartile range** | **Range** |
| --- | --- | --- | --- | --- |
| *Unit import cost (€ per unit)^*^* | | | | |
| Air freight | Shipment | 0.02 | .003–0.87 | 0.0004–22 |
|  | Standard cargo | 0.005 | 0.003–0.06 | 0.0004–5.90 |
|  | Cool cargo | 1.85 | 1.47–8.09 | 0.23–22.07 |
|  | Frozen cargo | 1.99 | 1.99–1.99 | 1.99–1.99 |
| Customs clearance | Shipment | 0.00008 | 0.00008–0.0007 | 0.00004–2.26 |
|  | Standard cargo | 0.00008 | 0.00008–0.00008 | 0.00004–0.90 |
|  | Cool cargo | 0.04 | 0.04–0.04 | 0.04–2.26 |
|  | Frozen cargo | 0.45 | 0.45–0.45 | 0.45–0.45 |
| Land freight | Shipment | 0.002 | 0.0003–0.08 | 0.00004–1.87 |
|  | Standard cargo | 0.0005 | 0.0003–0.006 | 0.00004–0.53 |
|  | Cool cargo | 0.16 | 0.16–0.69 | 0.02–1.87 |
|  | Frozen cargo | 0.09 | 0.09–0.09 | 0.09–0.09 |
| Total | Shipment | 0.02 | 0.004–1.14 | 0.0006–24 |
|  | Standard cargo | 0.007 | 0.003–0.07 | 0.0006–6.43 |
|  | Cool cargo | 2.16 | 1.80–8.98 | 0.29–24 |
|  | Frozen cargo | 2.53 | 2.53–2.53 | 2.53–2.53 |
|  | | | | |
| *Percentage unit import cost (% of unit price)*^†^ | | | | |
| Air freight | Shipment | 10 | 4.1–23 | 0.1–907 |
|  | Standard cargo | 11 | 4.2–27 | 0.1–907 |
|  | Cool cargo | 5.9 | 3.1–16 | 0.6–81 |
|  | Frozen cargo | 4.4 | 4.4 | 4.4 |
| Customs clearance | Shipment | 0.1 | 0.02–0.4 | 0.0001–205 |
|  | Standard cargo | 0.08 | 0.01–0.3 | 0.0001–205 |
|  | Cool cargo | 0.2 | 0.07–0.4 | 0.01–36 |
|  | Frozen cargo | 1.0 | 1.0 | 1.0 |
| Land freight | Shipment | 0.9 | 0.4–2.1 | 0.01–82 |
|  | Standard cargo | 1.0 | 0.4–2.4 | 0.01–82 |
|  | Cool cargo | 0.5 | 0.3–1.3 | 0.05–6.9 |
|  | Frozen cargo | 1.3 | 0.2–2.4 | 0.2–2.4 |
| Total | Shipment | 12 | 4.5–35 | 0.1–989 |
|  | Standard cargo | 12 | 4.6–35 | 0.1–989 |
|  | Cool cargo | 8.7 | 3.8–21 | 0.7–88 |
|  | Frozen cargo | 5.6 | 5.6 | 5.6 |

N = ^*^85 and ^†^83. Percentage import costs exclude a donated item and the frozen cargo part of a test kit that was split across cargos. Kruskal-Wallis tests reject equality of the unit import cost distribution across cargo types for air freight, customs clearance, land freight and total import costs (all P < 0.001). Equal distribution across cargo types was not rejected for percentage unit import costs (air freight P = 0.46, customs clearance P = 0.12, land freight P = 0.24, and total unit import cost P = 0.67).

**Table S3:** Unit import costs of medical supplies for a tuberculosis program in Uzbekistan, by pharmaceutical form of imported items

| **Group** | **Pharmaceutical form of item (N)** | **Median** | **Interquartile range** | **Range** |
| --- | --- | --- | --- | --- |
| *Unit import cost (€ per unit)^*^* | | | | |
|  | Fluid ≥100 ml (8) | 2.06 | 1.04–3.97 | 0.62–6.43 |
|  | Ointment (3) | 0.19 | 0.05–1.01 | 0.05–1.01 |
|  | Powder (4) | 0.15 | 0.10–0.15 | 0.06–0.16 |
|  | Fluid <100 ml (10) | 0.07 | 0.04–0.28 | 0.02–6.07 |
|  | Wipe (1) | 0.005 | 0.005–0.005 | 0.005–0.005 |
|  | Tablet (38) | 0.004 | 0.002–0.007 | 0.0006–0.04 |
|  | Capsule (5) | 0.003 | 0.002–0.005 | 0.001–0.007 |
| Cool cargo | Fluid <100 ml (1) | 2.61 | 2.61–2.61 | 2.61–2.61 |
|  | Test/Test kit (13) | 2.05 | 1.8–8.98 | 0.29–24 |
| Frozen cargo | Test/Test kit (2) | 2.53 | 2.53–2.53 | 2.53–2.53 |
|  | | | | |
| *Percentage unit import cost (% of unit price)*^†^ | | | | |
| Standard cargo | Fluid ≥100 ml (8) | 149 | 57.7–352 | 4.6–989 |
|  | Wipe (1) | 111 | 111–111 | 111–111 |
|  | Ointment (3) | 29.4 | 4.2–231 | 4.2–231 |
|  | Fluid <100 ml (10) | 13.2 | 9.9–31 | 4.6–104 |
|  | Tablet (38) | 11.4 | 5.0–23 | 0.2–319 |
|  | Powder (4) | 3.96 | 3.2–13 | 2.9–22 |
|  | Capsule (5) | 3.06 | 0.9–11 | 0.1–15 |
| Cool cargo | Fluid <100 ml (1) | 41.4 | 41–41 | 41–41 |
|  | Test/Test kit (12) | 7.58 | 3.4–19 | 0.7–88 |
| Frozen cargo | Test/Test kit (1) | 5.62 | 5.6–5.6 | 5.6–5.6 |

N = ^*^85 and ^†^83. Percentage import costs exclude a donated item and the frozen cargo part of a test kit that was split across cargos. For unit import cost, Kruskal-Wallis tests reject the equality of the distribution across the pharmaceutical form of items in the standard cargo (P < 0.001) but not in the cool cargo (P = 0.71). Within standard cargo items, tablets and capsules had significantly lower median unit import costs than fluids ≥100 ml (both Dunn tests P < 0.001) and fluids <100 ml (P < 0.001 and P = 0.024). Percentage unit cost were significantly different between capsules and fluids ≥100 ml (P < 0.017).

**Table S4:** Unit import costs of medical supplies for a tuberculosis program in Uzbekistan, by packaging of imported items

| **Group** | **Packaging of item (N)** | **Median** | **Interquartile range** | **Range** |
| --- | --- | --- | --- | --- |
|  | | | | |
| *Unit import cost (€ per unit)^*^* | | | | |
| Standard cargo | Fluid bag (3) | 2.70 | 0.64–6.43 | 0.62–6.43 |
|  | Bottle (8) | 1.27 | 0.59–3.97 | 0.23–6.07 |
|  | Tube (3) | 0.19 | 0.05–1.01 | 0.05–1.01 |
|  | Vial (4) | 0.15 | 0.10–0.15 | 0.06–0.16 |
|  | Ampulla (6) | 0.04 | 0.03–0.07 | 0.02–0.08 |
|  | Sachet (2) | 0.03 | 0.005–0.06 | 0.005–0.06 |
|  | Capsule/Tablet (43) | 0.004 | 0.002–0.007 | 0.0006–0.04 |
| Cool cargo | Vial (1) | 2.61 | 2.61–2.61 | 2.61–2.61 |
|  | Test/Test kit (13) | 2.05 | 1.80–8.98 | 0.29–24 |
| Frozen cargo | Test/Test kit (2) | 2.53 | 2.53–2.53 | 2.53–2.53 |
|  | | | | |
| *Percentage unit import cost (% of unit price)*^†^ | | | | |
| Standard cargo | Fluid bag (3) | 520 | 106–989 | 106–989 |
|  | Sachet (2) | 58 | 4.5–111 | 4.5–111 |
|  | Tube (3) | 29 | 4.2–231 | 4.2–231 |
|  | Ampulla (6) | 22 | 12–35 | 4.6–104 |
|  | Bottle (8) | 17 | 9.5–149 | 4.6–184 |
|  | Capsule/Tablet (43) | 11 | 3.3–18 | 0.1–319 |
|  | Vial (4) | 6.0 | 3.2–15 | 2.9–22 |
| Cool cargo | Vial (1) | 41 | 41–41 | 41–41 |
|  | Test/Test kit (12) | 7.6 | 3.4–19 | 0.7–88 |
| Frozen cargo | Test/Test kit (1) | 5.6 | 5.6–5.6 | 5.6–5.6 |

N = ^*^85 and ^†^83. Percentage import costs exclude a donated item and the frozen cargo part of a test kit that was split across cargos. For unit import cost, Kruskal-Wallis tests reject the equality of the distribution across the packaging of items in the standard cargo (P < 0.001) but not in the cool cargo (P = 0.71). Within standard cargo items, tablets/capsules had a significantly lower median unit import cost than fluid bags (Dunn test P = 0.040).

**Table S5:** Predictors of unit import cost of medical supplies for a tuberculosis program in Uzbekistan

| **Unit import cost (€)** | **Weight model** | **Price model** | **Form model (short)** | **Form model** | **Packaging model** |
| --- | --- | --- | --- | --- | --- |
| *Cargo type* | | | | | |
| Cool cargo | 0.6 (0.49 to 0.7)^***^ | 0.4 (−0.19 to 0.98) | 2.49 (2.06 to 2.92)^***^ | 1.42 (0.49 to 2.35)^**^ | 1.34 (0.42 to 2.25)^**^ |
| Frozen cargo | 1.01 (0.78 to 1.24)^***^ | 0.22 (−1.26 to 1.7) | 2.4 (1.37 to 3.43)^***^ | 1.33 (0.18 to 2.47)^*^ | 1.24 (0.13 to 2.35)^*^ |
| *Unit weight and price* | | | | | |
| Unit weight (g) | 0.98 (0.95 to 1.02)^***^ |  |  |  |  |
| Unit price (€) |  | 0.77 (0.59 to 0.94)^***^ |  |  |  |
| Pharmaceutical from |  |  |  |  |  |
| Fluid ≥100 ml |  |  | 2.29 (1.75 to 2.83)^***^ | 2.7 (2.35 to 3.04)^***^ |  |
| Ointment |  |  |  | 1.72 (1.19 to 2.25)^***^ |  |
| Powder |  |  |  | 1.49 (1.02 to 1.95)^***^ |  |
| Test/Test kit |  |  |  | 1.48 (0.51 to 2.45)^**^ |  |
| Fluid <100 ml |  |  |  | 1.4 (1.08 to 1.71)^***^ |  |
| Wipe |  |  |  | 0.14 (−0.76 to 1.03) |  |
| Capsule |  |  |  | −0.13 (−0.55 to 0.29) |  |
| *Item packaging* | | | | | |
| Fluid bag |  |  |  |  | 2.76 (2.27 to 3.25) |
| Bottle |  |  |  |  | 2.52 (2.21 to 2.84)^***^ |
| Tube |  |  |  |  | 1.74 (1.25 to 2.23)^***^ |
| Test/Test kit |  |  |  |  | 1.58 (0.62 to 2.53)^**^ |
| Vial |  |  |  |  | 1.5 (1.07 to 1.92)^***^ |
| Ampulla |  |  |  |  | 1.03 (0.67 to 1.38)^***^ |
| Sachet |  |  |  |  | 0.68 (0.09 to 1.28)^*^ |
| Constant | −2.28 (−2.32 to −2.24)^***^ | −1.08 (−1.3 to −0.86)^***^ | −2 (−2.18 to −1.81)^***^ | −2.4 (−2.54 to −2.26)^***^ | −2.42 (−2.54 to −2.29)^***^ |
| R-squared (%) | 98.6 | 70.3 | 70.5 | 89.7 | 91.1 |
| Adj. R-squared (%) | 98.5 | 69.1 | 69.4 | 88.4 | 90 |
| N | 85 | 83 | 85 | 85 | 85 |

Coefficient (95% Confidence Interval). ^*^P < 0.05, ^**^P < 0.01, ^***^P  < 0.001. Unit import cost, weight and price were log 10-transformed. Weight has been the allocation base for transport costs, which represented 99% of the total import cost. The price model excludes a donated item and the frozen cargo part of a test kit that was split across cargos.**Table S6:** Predictors of percentage unit import cost of medical supplies for a tuberculosis program in Uzbekistan

| **Unit import cost (€)** | **Weight model** | **Price model** | **Form model (short)** | **Form model** | **Packaging model** |
| --- | --- | --- | --- | --- | --- |
| *Cargo type* | | | | | |
| Cool cargo | 0.62 (0.49 to 0.75)^***^ | 0.4 (−0.19 to 0.98) | 1.08 (0.57 to 1.59)^***^ | 1.19 (0.31 to 2.06)^**^ | 1.25 (0.37 to 2.13)^**^ |
| Frozen cargo | 1.04 (0.7 to 1.38)^***^ | 0.22 (−1.26 to 1.7) | 0.95 (−0.26 to 2.15) | 1.13 (−0.1 to 2.36) | 1.2 (0.01 to 2.4)^*^ |
| *Unit weight and price* | | | | | |
| Unit weight (g) | 0.99 (0.94 to 1.05)^***^ |  |  |  |  |
| Unit price (€) | −1.02 (−1.07 to −0.96)^***^ | −0.23 (−0.41 to −0.06)^**^ | −0.45 (−0.6 to −0.3)^***^ | −0.77 (−0.92 to −0.62)^***^ | −0.84 (−0.98 to −0.7)^***^ |
| *Pharmaceutical from* | | | | | |
| Fluid ≥ 100 ml |  |  | 1.58 (1.11 to 2.05)^***^ | 2.31 (1.9 to 2.71)^***^ |  |
| Ointment |  |  |  | 1.44 (0.91 to 1.97)^***^ |  |
| Fluid<100ml |  |  |  | 1.13 (0.79 to 1.47)^***^ |  |
| Powder |  |  |  | 1.09 (0.59 to 1.59)^***^ |  |
| Test/Test kit |  |  |  | 0.98 (0.03 to 1.92)^*^ |  |
| Wipe |  |  |  | 0.35 (−0.5 to 1.19) |  |
| Capsule |  |  |  | −0.25 (−0.65 to 0.15) |  |
| Item packaging |  |  |  |  |  |
| Fluid bag |  |  |  |  | 2.58 (2.09 to 3.07)^***^ |
| Test/Test kit |  |  |  |  | 1.13 (0.16 to 2.1)^*^ |
| Bottle |  |  |  |  | 2.2 (1.79 to 2.61)^***^ |
| Tube |  |  |  |  | 1.55 (1.05 to 2.04)^***^ |
| Vial |  |  |  |  | 1.24 (0.77 to 1.7)^***^ |
| Ampulla |  |  |  |  | 0.92 (0.57 to 1.28)^***^ |
| Sachet |  |  |  |  | 0.64 (0.08 to 1.21)^*^ |
| Constant | −0.3 (−0.38 to −0.22)^***^ | 0.92 (0.7 to 1.14)^***^ | 0.55 (0.34 to 0.76)^***^ | −0.08 (−0.33 to 0.16) | −0.2 (−0.42 to 0.02) |
| R-squared (%) | 95.4 | 9.1 | 42.6 | 71.6 | 74.5 |
| Adj. R-squared (%) | 95.1 | 5.7 | 39.6 | 67.7 | 70.9 |
| N | 83 | 83 | 83 | 83 | 83 |

Coefficient (95% Confidence Interval). ^*^P < 0.05, ^**^P < 0.01, ^***^P  < 0.001. Unit import cost, weight and price were log 10-transformed. Weight has been the allocation base for transport costs, which represented 99% of the total import cost. Model exclude a donated item and the frozen cargo part of a test kit that was split across cargos.

**Figure S1:** Unit import costs of medicines imported as standard cargo by a tuberculosis program in Uzbekistan, by item

|  |
| --- |
|  |

Air freight Customs clearance Land freight

N = 69.**Figure S2:** Percentage unit import costs of medicines imported as standard cargo by a tuberculosis program in Uzbekistan, by item

|  |
| --- |
|  |

Air freight Customs clearance Land freight

N = 69.

**Figure S3:** Distribution of the unit import costs in a tuberculosis program in Uzbekistan

(a) Unit import cost

(b) Unit air freight cost

(c) Unit customs clearance costs

(d) Unit land freight cost

Standard cargo Cool cargo Frozen cargo

N = 85. Logarithmic x-axis. Normal distributions of unit import, air freight, and land freight costs on a decadic logarithm scale are not rejected for cool cargo (Shapiro-Wilk test P = 0.17, P = 0.41, and P = 0.41). The frozen cargo contained not enough items to test its unit import cost distribution.

**Figure S4:** Distribution of the percentage unit import costs in tuberculosis program in Uzbekistan

(a) Percentage unit import cost

(b) Percentage unit air freight cost

(c) Percentage unit customs clearance costs

(d) Percentage unit land freight cost

Standard cargo Cool cargo Frozen cargo

N = 83. Logarithmic x-axis. Normal distributions of percentage unit import, air freight, customs clearance, and land freight costs on a decadic logarithm scale are not rejected for standard cargo (all four Shapiro-Wilk tests P > 0.4), cool cargo (P > 0.1), and frozen cargo (P > 0.4). The frozen cargo contained not enough items to test its unit import cost distribution.

**Figure S5:** Unit import costs of medical supplies for a tuberculosis program in Uzbekistan, by packaging of imported items

(a) Unit import costs^*^

(b) Percentage unit import costs^†^

**Median**

--- Standard cargo --- Cool cargo --- Frozen cargo --- Shipment

N = ^*^85 and ^†^83. Logarithmic y-axis. Tablets Capsule/Tablet (43 items), test or test kit materials (15 items), bottle (8 items), ampulla (6 items), vial (5 items), fluid bag (3 items), tube (3 items), and sachet (2 items).
